# A Comparative Analysis of Universal and Sentinel Surveillance Data for Coronavirus Disease 2019: Insights From Argentina, Chile, and Mexico (2020–2022)

**DOI:** 10.1093/infdis/jiae620

**Published:** 2025-03-10

**Authors:** Lidia Redondo-Bravo, Kinda Zureick, Carla Voto, Xaviera Molina Avendaño, Laura Flores-Cisneros, Ashley Fowlkes, Luciana Eva Iummato, Carlos Giovacchini, Maria Fernanda Olivares Barraza, Paula Rodriguez Ferrari, Rosaura Gutiérrez-Vargas, Christian Arturo Zaragoza-Jiménez, Gabriel García-Rodríguez, Hugo López-Gatell, Ángel Rodríguez, Paula Couto, Marc Rondy, Andrea S Vicari

**Affiliations:** PAHO Health Emergencies, Pan American Health Organization, Washington, DC, USA; Influenza Division, US Centers for Disease Control and Prevention, Atlanta, Georgia, USA; Dirección de Epidemiología, Ministerio de Salud, Buenos Aires, Argentina; Oficina de País de Chile, Organización Panamericana de la Salud, Santiago de Chile, Chile; Centro de Epidemiología y Políticas de Salud, Universidad del Desarrollo de Chile, Santiago de Chile, Chile; Oficina de País de México, Organización Panamericana de la Salud, Ciudad de México, México; Influenza Division, US Centers for Disease Control and Prevention, Atlanta, Georgia, USA; Dirección de Epidemiología, Ministerio de Salud, Buenos Aires, Argentina; Dirección de Epidemiología, Ministerio de Salud, Buenos Aires, Argentina; Departamento de Epidemiología, Ministerio de Salud, Santiago de Chile, Chile; Departamento de Epidemiología, Ministerio de Salud, Santiago de Chile, Chile; Dirección de Información Epidemiológica, Dirección General de Epidemiología, Secretaría de Salud, Ciudad de México, México; Dirección de Información Epidemiológica, Dirección General de Epidemiología, Secretaría de Salud, Ciudad de México, México; Dirección de Información Epidemiológica, Dirección General de Epidemiología, Secretaría de Salud, Ciudad de México, México; Subsecretaría de Prevención y Promoción de la Salud, Secretaría de Salud, Ciudad de México, México; PAHO Health Emergencies, Pan American Health Organization, Washington, DC, USA; PAHO Health Emergencies, Pan American Health Organization, Washington, DC, USA; PAHO Health Emergencies, Pan American Health Organization, Washington, DC, USA; PAHO Health Emergencies, Pan American Health Organization, Washington, DC, USA

**Keywords:** SARS-CoV-2 surveillance, universal surveillance, sentinel surveillance, COVID-19 pandemic, respiratory viruses

## Abstract

**Background:**

In 2020, countries implemented universal surveillance to detect and monitor severe acute respiratory syndrome coronavirus 2 (SARS-CoV-2) cases. Although crucial for early monitoring efforts, universal surveillance is resource intensive. To understand the implications of transitioning from universal to sentinel surveillance for monitoring SARS-CoV-2 transmissibility, morbidity and mortality, and disease seriousness, we compared measures of SARS-CoV-2 reported from both surveillance strategies in Argentina, Chile, and Mexico.

**Methods:**

We obtained weekly case counts in Argentina, Chile, and Mexico, in periods when both universal and sentinel surveillance were ongoing. To assess the countries' surveillance strategies, we measured the proportion of total sites that were included in sentinel surveillance. We compared 8 measures of SARS-CoV-2 transmissibility, morbidity and mortality, and disease seriousness between sentinel and universal surveillance and assessed the correlation between the 2 strategies for the 8 measures. Pearson and Spearman correlation was classified as very strong (*r*_s_ = 0.8–1.0), strong (*r*_s_ = 0.60–0.79), moderate (*r*_s_ = 0.50–0.59), or poor (*r* < 0.50).

**Results:**

The proportion of total sites included in sentinel surveillance was 5.8% for Argentina, 1.1% for Chile, and 7.6% for Mexico. A total of 21 measures were calculated (8 for Mexico, 8 for Chile, and 5 for Argentina). Of these, 17 showed consistency between the 2 surveillance strategies, with strong or very strong correlations (*r* = 0.66–0.99): all 8 measures for Mexico, 6 of 8 measures for Chile, and 3 of 5 measures for Argentina. Each country had ≥1 measure reflecting transmissibility and ≥1 reflecting morbidity and mortality for which the correlation was strong or very strong. Chile and Mexico also had ≥1 measure of disease seriousness for which the correlation was strong.

**Conclusions:**

Our findings suggest that the integration of SARS-CoV-2 into national sentinel surveillance can yield information comparable to that provided by nationwide universal surveillance for measures related to SARS-CoV-2 transmissibility, morbidity and mortality, and seriousness of disease.

With >6.9 million deaths due to coronavirus disease 2019 (COVID-19) worldwide since December 2019, the global impact of COVID-19 on both the public health and the economy has demonstrated the critical need for robust disease surveillance systems [1]. In 2020, the World Health Organization (WHO) recommended universal testing to limit the spread of severe acute respiratory syndrome coronavirus 2 (SARS-CoV-2) through case finding and contact tracing [2]. As a result, most countries in the region of the Americas implemented comprehensive (universal) COVID-19 surveillance strategies. In some cases, this universal surveillance coexisted with sentinel surveillance of influenza and other respiratory viruses, with the inclusion of SARS-CoV-2. This led to a duplication of efforts for monitoring trends, which became highly relevant in 2022 when many countries ceased isolation and quarantine practices.

The countries in the region of the Americas have a long history of respiratory virus surveillance. Since the launching of the Global Influenza Surveillance and Response System >70 years ago, respiratory virus surveillance has predominantly focused on influenza for early detection of new pandemic strains, monitoring influenza circulation, and determining influenza vaccine composition [3, 4]. To monitor influenza circulation and select vaccine strains, WHO recommends that countries use a sentinel surveillance approach. Sentinel surveillance involves systematically collecting epidemiological and virological data from a subset of healthcare providers and hospitals, targeting patients meeting case definitions for influenzalike illness (ILI) or severe acute respiratory infection (SARI) [5]. In the Region of the Americas, since 2014, SARI sentinel surveillance has been strengthened by the SARInet plus network, which focuses on improving the monitoring, detection, and reporting of respiratory diseases, particularly those with pandemic potential. SARInet encourages data sharing and collaboration and provides training, capacity building, and tools for countries to improve their surveillance capabilities [6, 7].

In 2020, during the acute phase of the COVID-19 pandemic, and following WHO recommendations [2], countries implemented universal surveillance to detect and monitor all COVID-19 cases, in order to isolate them and follow their contacts, reduce transmissibility, and provide critical epidemiological data to inform public health measures. However, although crucial for early monitoring efforts, universal surveillance is resource intensive. In March 2022, the Pan American Health Organization (PAHO) and WHO conducted consultations to advise on directions for COVID-19 surveillance [2, 8–10]. These consultations concluded that integrating SARS-CoV-2 into existing sentinel surveillance systems for respiratory viruses could provide a viable approach for monitoring viral and epidemiological characteristics but stressed the need to generate more evidence supporting this transition [2, 8–10].

To better understand the implications of transitioning from universal to sentinel surveillance on monitoring and characterizing SARS-CoV-2 epidemiology, we compared measures of SARS-CoV-2 transmissibility, morbidity and mortality, and seriousness of the disease reported from universal surveillance with those reported from sentinel surveillance in Argentina, Chile, and Mexico between 2020 and 2022.

## METHODS

### Data Sources

Data from Argentina, Chile, and Mexico's universal and sentinel surveillance systems were included in this study. We selected these PAHO member states based on 2 criteria: (1) countries that maintained sentinel surveillance throughout the duration or during ≥6 months of the COVID-19 pandemic and (2) countries that had different proportions of sentinel site coverage within the national healthcare system relative to universal surveillance. Mexico had a high coverage proportion for both sentinel ILI and SARI sites, while Argentina and Chile had intermediate coverage proportions. Among these, Argentina had greater coverage of ILI sites, whereas Chile had greater coverage of SARI sites. Universal surveillance systems aimed at capturing every suspected COVID-19 case according to national case definitions tested in a health facility, nationwide. Sentinel surveillance systems aimed at capturing a subset of ILI and/or SARI cases following each country's established case definitions tested in strategically selected healthcare settings [11] (Supplementary Methods).

The study periods varied by country and were limited to the periods during which both universal and sentinel surveillance were ongoing. The study included data through the end of 2022, when countries started analyzing their results.

We obtained aggregate data for weekly case counts from each country's national internal and public-facing databases. Each country used different dates as reference on the basis of the date used in their national surveillance reports. For example, weekly case numbers were reported using the date of symptom onset in Chile and the reporting date in Mexico. In Argentina, the date of reference was the first date with data available in the following sequence: date of symptom onset, date of consultation, date of sample collection, or date of case notification.

### Measures

To assess the countries' surveillance systems, we measured the proportion of total sites that were included in sentinel surveillance by dividing the number of sentinel sites by the number of sites included in the universal surveillance overall, in outpatient clinics, and in hospitals. We compared 8 measures of SARS-CoV-2 transmissibility, morbidity and mortality, and seriousness of disease between sentinel and universal surveillance based on WHO's pandemic influenza severity assessment (PISA) measures (Table 1) [12]. Measures of transmissibility included percentage and number of specimens positive for SARS-CoV-2 from any setting and number of cases positive for SARS-CoV-2 from outpatient settings. Measures that represented morbidity and mortality were number of hospitalizations with a positive test result for SARS-CoV-2, number of hospitalized cases with a positive SARS-CoV-2 result requiring admission to an intensive care unit (ICU), and number of hospitalizations with a positive SARS-CoV-2 result ending in death. Measures of seriousness of disease included percentage of hospitalized cases with a positive SARS-CoV-2 result requiring ICU admission and percentage of hospitalizations with a positive SARS-CoV-2 result ending in death. Measures not available for both surveillance strategies were excluded from the country-level analysis.

**Table 1.** Measures of Transmissibility, Morbidity and Mortality, and Seriousness of Disease With Universal and Sentinel Surveillance Strategies

Measure	Calculation for Universal Surveillance Strategy^a^	Calculation for Sentinel Surveillance Strategy^b^
Transmissibility measures		
1. SARS-CoV-2 positivity (%)	% of tested cases positive for SARS-CoV-2	% of SARI and ILI/ARI tested cases positive for SARS-CoV-2
2. Total cases (no.)	No. of new cases positive for SARS-CoV-2	No. of new SARI and ILI/ARI cases positive for SARS-CoV-2
3. Outpatient cases (no.)	No. of new outpatient cases positive for SARS-CoV-2	No. of new ILI/ARI cases positive for SARS-CoV-2
Morbidity and mortality measures		
4. Hospitalized cases (no.)	No. of new SARS-CoV-2–positive hospitalized cases	No. of new SARS-CoV-2–positive SARI hospitalized cases
5. ICU admissions (no.)	No. of new SARS-CoV-2–positive hospitalized cases requiring ICU admission	No. of new SARS-CoV-2–positive SARI cases requiring ICU admission
6. Deaths (no.)	No. of new deaths among SARS-CoV-2–positive hospitalized cases	No. of new deaths among SARS-CoV-2–positive SARI cases
Seriousness of disease measures		
7. ICU admissions (%)	% of SARS-CoV-2–positive hospitalized cases requiring ICU admission	% of SARS-CoV-2–positive SARI cases requiring ICU admission
8. Deaths (%)	% of deaths among hospitalized SARS-CoV-2 cases	% of deaths among SARS-CoV-2–positive SARI cases

Abbreviations: ARI, acute respiratory infection; ICU, intensive care unit; ILI, influenzalike illness; SARI, severe acute respiratory infection; SARS-CoV-2, severe acute respiratory syndrome coronavirus 2.

^a^Cases identified via universal surveillance were based on each country's respective coronavirus disease 2019 case definition.

^b^Cases identified via sentinel surveillance were based on each country's respective ILI, ARI, or SARI case definitions.

### Statistical Analysis

We analyzed the correlation between universal and sentinel surveillance systems for the 8 measures by week at the national level. We used Pearson's (*r*) and Spearman's (*r*_s_) correlation coefficients for linear and nonlinear correlation, respectively (evaluated by scatterplot visual inspection) [13]. The correlation was classified as very strong (*r*_s_ = 0.8–1.0), strong (*r*_s_ = 0.60–0.79), moderate (*r*_s_ = 0.50–0.59), or poor (*r*_s_ < 0.50) (Table 2) [13]. For each measure, we analyzed the correlation between the proportion of total healthcare facilities in sentinel surveillance systems and the strength of previously determined correlation.

**Table 2.** Correlation Coefficient Cutoff Values

Cutoff Values	Interpretation
0.80–1.0	Very strong
0.60–0.79	Strong
0.50–0.59	Moderate
<0.50	Poor

To determine whether the transition from sentinel to universal surveillance could have an impact on information timeliness, we explored the temporal relationship between indicators. We conducted a cross-correlation analysis to assess the relationship between sentinel and universal surveillance data for each indicator across various lagged time points. The correlation coefficient was calculated at each lag, quantifying the degree of association between the 2 surveillance strategies for that specific time shift. Positive coefficients indicate a direct relationship between sentinel and universal data, while negative coefficients suggest an inverse relationship., The magnitude of the coefficient reflects the strength of the relationship at a given lag. The lag at which the cross-correlation function peaks (either positively or negatively) identifies the lead-lag relationship between sentinel and universal data, indicating whether one strategy precedes or follows the other in capturing trends for a particular indicator.

Microsoft Excel and RStudio version 1.4.1103 software were used for data management and analysis. Tableau 2020.4 software was used for graphic design.

This study was reviewed by the institutional review boards at the US Centers for Disease Control and Prevention (STARS project determination no. 0900f3eb81f7597b) and each of the participant countries, and it was determined to be exempt from human subjects review because of the use of aggregated case counts obtained from surveillance data, which ensured the anonymity of individuals.

## RESULTS

The study periods assessed in this study were 5 June to 26 December 2022 for Argentina, 3 March 2020 to 10 October 2022 for Chile (8 May to 10 October 2022 for outpatient data), and 28 February 2020 to 12 September 2022, for Mexico. These periods were selected because they represent the time frames during which both sentinel and universal surveillance strategies were operational within each country.

In Argentina, universal surveillance for SARS-CoV-2 included 1026 sites, 616 outpatient clinics, 410 hospitals, and 506 laboratories. Sentinel surveillance included 60 sites, 57 outpatient clinics (ILI sites), 3 hospitals (SARI sites), and 55 testing laboratories. The proportions of healthcare facilities included in the sentinel strategy were 5.8% in total, 9.3% for outpatient clinics, and 0.7% for hospitals (Table 3).

**Table 3.** Key Characteristics of the Universal and Sentinel Surveillance Strategies in Argentina, Chile, and Mexico, 2020–2022

Characteristic	Argentina	Chile	Mexico
Study period	5 Jun–26 Dec 2022	3 Mar 2020–10 Oct 2022^a^	28 Feb 2020–12 Sep 2022
Universal surveillance sites, no.	1026	4380	6253^b^
Outpatient clinics	616	4151	6253^b^
Hospitals	410	229	1519
Testing laboratories	506	213	183
Sentinel surveillance sites, no.	60	50	473^b^
Outpatient clinics	57	41	473^b^
Hospitals	3	9	362
Testing laboratories	55	11	183
Sentinel sites as proportion of healthcare facilities (universal surveillance sites), %	5.8	1.1	7.6
Outpatient clinics	9.3	1.0	7.6
Hospitals	0.7	3.9	23.8
Testing laboratories	10.9	5.2	100.0

^a^Chile outpatient data include cases from 8 May to 10 October 2022.

^b^In Mexico, in addition to outpatient clinics, some hospitals participate in the surveillance of outpatient cases through their emergency departments.

In Chile, universal surveillance for SARS-CoV-2 included 4380 sites, 4151 outpatient clinics, 229 hospitals, and 213 laboratories. Sentinel surveillance included 50 sites, 41 ILI sites, 9 SARI sites, and 11 testing laboratories. The proportions of healthcare facilities included in the sentinel strategy were 1.1% in total, 1.0% for outpatient clinics, and 3.9% for hospitals (Table 3).

In Mexico, universal surveillance for SARS-CoV-2 included 6253 sites, 6253 outpatient clinics and hospital emergency units for outpatients, 1519 hospitals for inpatients, and 183 testing laboratories. Sentinel surveillance included 473 sites, 111 outpatient ILI sites, 362 hospital-based ILI and SARI sites, and 183 testing laboratories. The proportions of healthcare facilities included in the sentinel strategy were 7.6% in total, 7.6% for outpatient clinics, and 23.8% for hospitals (Table 3).

### Transmissibility Measures

**Figure 1.**
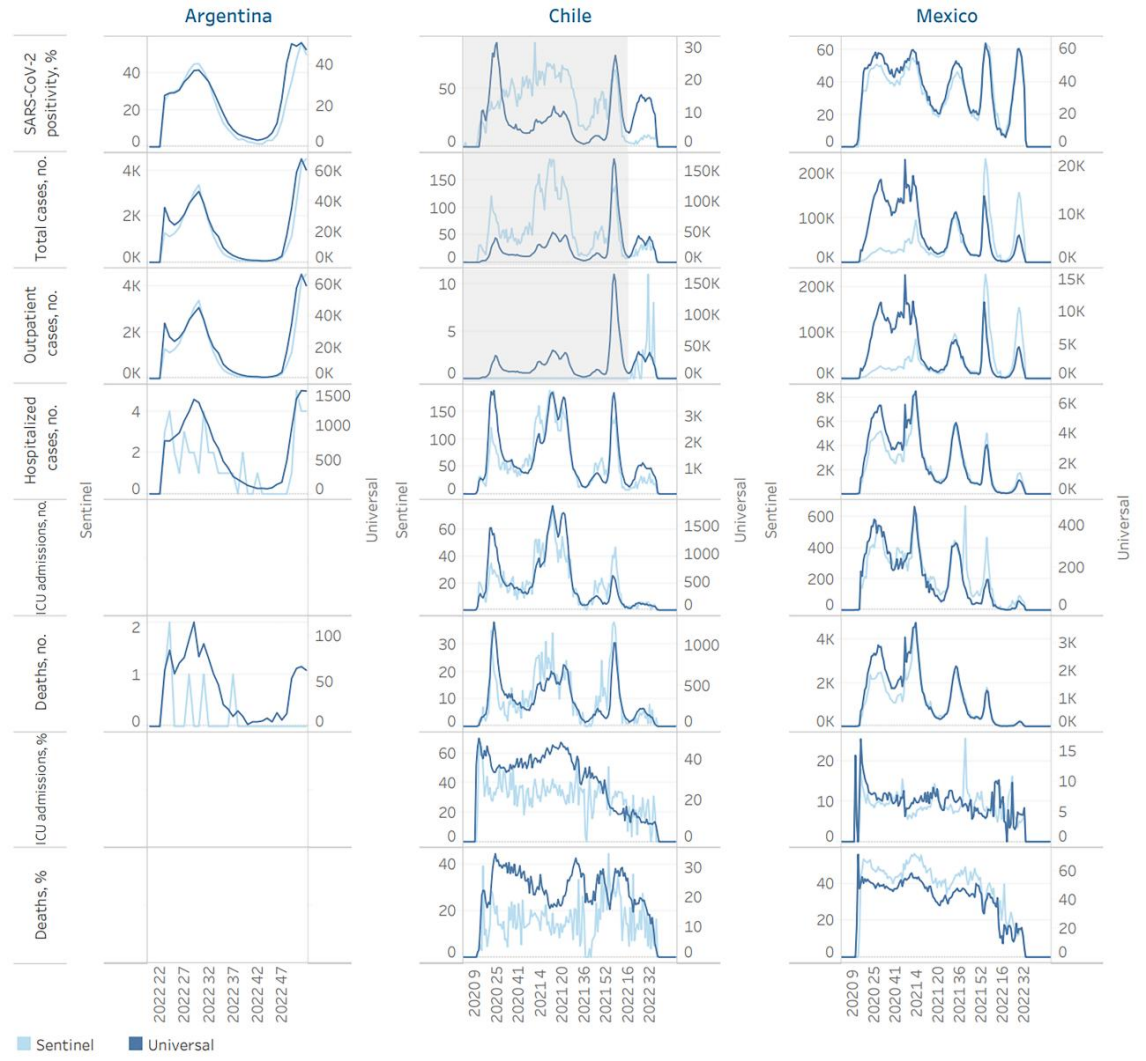
Epidemiological curves of the parameters corresponding to sentinel and universal surveillance of severe acute respiratory syndrome coronavirus 2 (SARS-CoV-2) by epidemiological week by country. The percentage of SARS-CoV-2 positivity and the number of total cases include both inpatient and outpatient cases. Gray shaded areas represent periods during which only inpatient cases were captured using the sentinel strategy, as Chile outpatient data included cases only from 8 May to 10 October 2022. The x-axis represents the year and the corresponding epidemiological week. Abbreviation: ICU, intensive care unit.

In Argentina transmissibility measures were very strongly correlated between sentinel and universal surveillance systems (*r*_s_ = 0.98–0.99). In Chile, 2 of 3 transmissibility indicators (counts of outpatient cases and percentage positivity for SARS-CoV-2) were poorly correlated (*r*_s_ < 0.5) and total case counts were strongly correlated (*r*_s_ = 0.72) between the 2 systems. In Mexico, all transmissibility measures were strongly or very strongly correlated (*r*_s_ = 0.76–0.98) between the 2 systems (Figure 1, Table 4, and Supplementary Figure 1).

### Morbidity and Mortality Measures

In Argentina, counts of hospitalized cases showed a very strong correlation (*r*_s_ = 0.85) between sentinel and universal surveillance systems, whereas counts of deaths were poorly correlated between the 2 systems (*r*_s_ < 0.5). In Chile, all morbidity and mortality measures showed correlations ranging from moderate to very strong (*r*_s_ > 0.5). Specifically, measures with a very strong correlation included counts of hospitalized cases, ICU admissions, and fatal cases (*r*_s_ = 0.80–0.89). In Mexico, correlations were very strong for all measures (*r*_s_ = 0.90–0.97) (Figure 1, Table 4, and Supplementary Figure 1).

**Table 4.** Correlation Between Sentinel and Universal Surveillance Strategies for 8 Measures of Transmissibility, Morbidity and Mortality, and Seriousness of Disease in Argentina, Chile, and Mexico

Surveillance Outcome	Measure	Argentina	Chile	Mexico
Transmissibility	1. SARS-CoV-2 positivity (%)	0.99	0.45	0.98
	2. Total cases	0.98	0.72	0.76
	3. Outpatient cases	0.98	0.09^a^	0.76
Morbidity and mortality	4. Hospitalized cases	0.85^b^	0.89^b^	0.97
	5. ICU admissions	NA	0.88	0.90
	6. Deaths	0.41^b^	0.80	0.96
Seriousness of disease	7. ICU admissions (%)	NA	0.66	0.70
	8. Deaths (%)	NA	0.59	0.93

Abbreviations: ICU, intensive care unit; NA, not assessed; SARS-CoV-2, severe acute respiratory syndrome coronavirus 2.

^a^Chile outpatient data include cases from 8 May to 10 October 2022.

^b^Spearman ρ coefficients.

### Seriousness of the Disease Measures

In Argentina, no measures of disease seriousness were calculated due to small sample size and limited data availability. In Chile, the proportion of ICU admissions among hospitalized cases with a positive SARS-CoV-2 result showed a strong correlation between sentinel and universal surveillance systems (*r*_s_ = 0.66), while the proportion of deaths among hospitalized cases with a positive SARS-CoV-2 result showed a moderate correlation (*r*_s_ = 0.59). In Mexico, the proportion of ICU admissions showed a strong correlation between the 2 systems (*r*_s_ = 0.7), while the proportion of deaths among hospitalized cases showed a very strong correlation (*r*_s_ = 0.93) (Figure 1, Table 4, and Supplementary Figure 1).

### Correlation Coefficients and Sentinel Strategy Coverage

**Figure 2.**
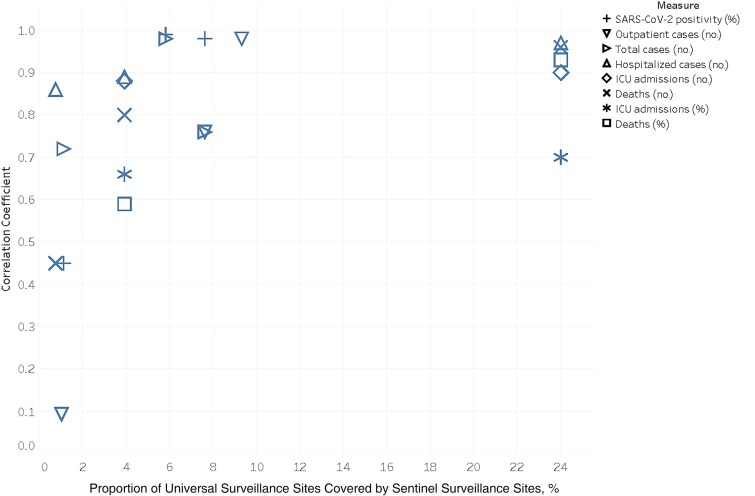
Scatterplot comparison between correlation coefficients and sentinel site coverage strategy in Argentina, Chile, and Mexico. Abbreviation: ICU, intensive care unit.

There was strong correlation (*r*_s_ = 0.66) between the correlation coefficients for each set of parameters and the percentage of universal sites included in sentinel surveillance. Of all the measures evaluated across the 3 countries at the national level, 4 exhibited a weak correlation: death counts and the percentage of deaths among hospitalized cases in Argentina (corresponding to a 0.7% sentinel site coverage of hospitals), and SARS-CoV-2% positivity and outpatient cases in Chile (corresponding to a sentinel site coverage of 1.0%) (Figure 2).

### Lag Analysis

In Argentina, the cross-correlation function for death counts peaked at lag +5, indicating a 5-week delay in reporting death counts from sentinel surveillance compared to universal surveillance. No lag was observed for the other measures. In Chile, the cross-correlation function for total case counts peaked at lag +1, revealing a 1-week delay in reporting total SARS-CoV-2 cases from sentinel data compared with universal data. In addition, a 4-week delay was observed in reporting outpatient SARS-CoV-2 cases with sentinel data relative to universal data. In Mexico, the cross-correlation function for both the percentage positivity and the total case counts peaked at lag +1, identifying a 1-week lag in the values reported with sentinel data compared with those with universal data for both measures. (Supplementary Figure 2).

## DISCUSSION

The COVID-19 pandemic highlighted a challenging context for respiratory virus surveillance, with 2 parallel strategies coexisting in many countries. This overlap led to duplication of some primary objectives and resources, emphasizing the need for a more integrated and sustainable approach that maintains the capacity to monitor the epidemiological characterization of SARS-CoV-2 [8–10]. Using data from 3 countries with differing approaches to their sentinel surveillance structure, we found that sentinel and universal surveillance systems were correlated strongly with key measures of SARS-CoV-2 transmissibility, morbidity and mortality, and disease seriousness. Considering the 8 measures assessed in each country, 17 of the total 21 measures were consistent between the 2 surveillance strategies, differing in accordance with the country's approach. Each country had ≥1 measure reflecting transmissibility and ≥1 reflecting morbidity and mortality for which the correlation was strong or very strong. Chile and Mexico also had ≥1 measure of diseases seriousness for which the correlation was strong. These findings indicate that sentinel surveillance can accurately depict trends in transmissibility and morbidity and mortality.

The findings from this study suggest a relationship between the proportion of sites covered by sentinel surveillance and the observed correlations between universal and sentinel surveillance data. For example, in Chile, which has invested substantially in hospital-based sentinel sites, measures of morbidity and mortality and disease seriousness were strongly correlated, while outpatient transmissibility measures were less strong. In contrast, in Argentina, where outpatient clinics are more represented than hospitals in the sentinel strategy, correlation coefficients for transmissibility measures were very strong. Moreover, at the national level, the 4 measures that exhibited poor correlation were associated with sentinel strategies with <2% site coverage. However, the observed correlations might not only be explained by sentinel coverage.

Variations in population coverage among sites should be considered, as these can vary widely between countries due to differences in the number of beds per site. Moreover, correlations tend to be weaker for infrequent events such as deaths or ICU admissions, potentially indicating lower reliability of sentinel surveillance data for these measures compared to universal surveillance data. Some measures from sentinel strategies with <2% site coverage still demonstrated good correlation coefficients, probably because of differences in population size covered by sentinel sites. Healthcare-seeking behavior patterns may have been modified during the pandemic and could have influenced the data collected [14–16]. However, these changes in behavior likely affected sites from both universal and sentinel surveillance strategies equally, thus not affecting the overall correlation.

Altogether, our correlation findings suggest that transitioning from universal to sentinel surveillance would not have a substantial impact on monitoring of SARS-CoV-2 transmissibility, morbidity and mortality, and seriousness of the disease. From a temporal perspective, either no lags or a 1-week lag were detected for those measures showing moderate to very strong correlation. These findings align with recommendations from consultations led by PAHO and WHO in 2022, which emphasized sentinel surveillance as a core approach for monitoring the epidemiological and virological characteristics of respiratory viruses on a routine basis. Although evidence is scarce, these findings also align with those of a few studies that compare sentinel versus universal surveillance in the research literature [17, 18].

While universal surveillance offers potential benefits beyond trend monitoring—such as early case detection, enabling timely treatment and isolation to reduce transmission—our findings suggest that transitioning to sentinel surveillance earlier during a pandemic may provide a sustainable and efficient alternative for ongoing surveillance. The observation that sentinel systems with <2% site coverage can still align with universal data underscores the effectiveness of a well-selected small number of representative sites for monitoring population trends. This highlights the potential for sentinel strategies to serve as a resource-efficient approach, particularly once correlations with universal surveillance are established. Future studies should further explore the balance between the initial advantages of universal surveillance and the long-term sustainability of sentinel systems, especially in resource-limited settings and varying pathogen scenarios.

This study was done in 3 countries that vary significantly in their approaches to implementing sentinel surveillance, which suggests that the conclusion could be applied to a variety of countries in the region. For countries lacking the capacity for extensive surveillance, the conclusions drawn from other countries can serve as valuable insights to adapt and apply within their respective systems. It is worth highlighting that, when comparing sentinel surveillance with universal surveillance at the subnational level, countries might need to be cautious, given the fact that correlation can worsen with small numbers of cases.

This study has several limitations. First, to understand the level of representativeness of sentinel sites, we used the percentage of universal sites that were also designated as sentinel sites. A more accurate measure would have been to include the population coverage of sentinel sites; however, these data are lacking in many countries in the Americas, including those in this study. This limitation may affect the interpretation of results, particularly for indicators with a low number of events (eg, ICU admissions and deaths), where weaker correlations could be influenced by limited site representativity. Future studies should incorporate population coverage data to better evaluate the representativeness of sentinel systems and their impact on the reliability of surveillance data, especially for rare events. Second, variations in the epidemiological situation across populations covered by sentinel sites may have influenced the comparability of data between sentinel and universal surveillance strategies. Differences in the spread of SARS-CoV-2 among these populations highlight the need to consider both coverage and representativity, as well as the dynamic nature of epidemiological trends, when interpreting surveillance data.

Third, variations in case definitions between countries' sentinel and universal strategies (Supplementary Methods) may limit the generalizability of these results. More sensitive case definitions can identify a higher number of cases but tend to be less specific, resulting in larger and more stable sample sizes. Conversely, more specific definitions reduce costs but produce smaller sample sizes and more variable trends. Universal surveillance used a broader case definition for COVID-19 than sentinel surveillance, which could have influenced the sample size in addition to the number of sites [5, 19–21]. However, this study focuses on the consistency of surveillance data between 2 strategies within each country, rather than the comparison of case definitions across countries. Finally, although differences in time periods between countries could result in variations in outcomes, our approach is designed to interpret these variations in relation to the surveillance systems implemented within each national context, avoiding direct cross-country comparisons. This emphasis ensures that the results reflect the strengths and limitations of each surveillance strategy within its specific context, rather than being confounded by structural and temporal heterogeneity across countries.

In conclusion, our findings suggest that the integration of SARS-CoV-2 into national sentinel surveillance can yield information comparable to that provided by nationwide universal surveillance for measures related to SARS-CoV-2 transmissibility, morbidity and mortality, and seriousness of disease. Although sentinel systems may not capture the majority of cases, countries that maintained a consistent system throughout the COVID-19 pandemic were able to effectively use these systems for trend monitoring of SARS-CoV-2 and studying the epidemiology of cases in a sustainable way. Finally, the methods proposed in this study facilitate comparisons between universal and sentinel surveillance strategies for SARS-CoV-2, providing guidance for decision making in transitioning from one strategy to another.

## Supplementary Data

Supplementary materials are available at *The Journal of Infectious Diseases* online (http://jid.oxfordjournals.org/). Supplementary materials consist of data provided by the author that are published to benefit the reader. The posted materials are not copyedited. The contents of all supplementary data are the sole responsibility of the authors. Questions or messages regarding errors should be addressed to the author.

## Supplementary Material

jiae620_Supplementary_Data

## Notes

***Acknowledgments.*** We extend our sincere gratitude to Wilmer Marquiño, Mario Cruz Peñate, and Jean Marc Gabastou from PAHO country offices in Argentina, Chile, and Mexico for their exceptional contributions to the successful execution of this project in the participating countries. We also thank Olaya Astudillo for her invaluable support and guidance in reviewing and editing this manuscript. Their expertise and dedication were instrumental in the development and completion of our research.

***Author contributions*.** L. R. B. conceived the study and designed the research methods. C. J. V., X. M. A., and L. F. C. collected the data. L. R. B., C. J. V., X. M. A., and L. F. C. analyzed the data. L. R. B., K. Z., C. J. V., X. M. A., L. F. C., A. F., L. E. I., C. M. G., M. F. O. B., P. R. F., R. G. V., C. A. Z. J., G. G. R., H. L. G., A. R., P. C., and M. R. contributed to the theoretical framework and literature review. L. R. B., K. Z., A. F., and M. R. drafted the manuscript. C. J. V., X. M. A., L. F. C., L. E. I., C. M. G., M. F. O. B., P. R. F., R. G. V., C. A. Z. J., G. G. R., H. L. G., A. R., P. C., and A. V. provided critical revisions. All authors interpreted the results, and all authors reviewed and approved the final version of the manuscript.

***Financial support*.** This work was supported by the US Centers for Disease Control and Prevention, which collaborated as part of the project for this study.

***Supplement sponsorship*.** This article appears as part of the supplement “Advancing Respiratory Virus Surveillance and Preparedness in the Americas: A Decade of SARInet's Impact,” sponsored by the Pan American Health Organization.

***Potential conflicts of interest*.** All authors: No reported conflicts. All authors have submitted the ICMJE Form for Disclosure of Potential Conflicts of Interest. Conflicts that the editors consider relevant to the content of the manuscript have been disclosed.
