# Supplementary material for: A Comparative Analysis of Universal and Sentinel Surveillance Data for Coronavirus Disease 2019: Insights From Argentina, Chile, and Mexico (2020–2022)
Source: J Infect Dis. 2025 Mar 10;231(Suppl 2):S114–22. doi: 10.1093/infdis/jiae620 (PMC11892001; doi:10.1093/infdis/jiae620)
Supplement: jiae620_Supplementary_Data [file jiae620_supplementary_data.zip › Universal_Sentinel_Surveillance_Appendix.docx]

**Supplementary Materials**

**Supplementary Methods**

***Data Sources: Case Definitions by Country***

1. **Argentina**

***Universal Surveillance of Confirmed COVID-19 Cases:***

Any confirmed case of COVID-19 through etiological diagnosis. Since April 2022, the surveillance strategy directs etiological diagnosis for SARS-CoV-2 infection in outpatient settings for individuals aged over 50 years, those with risk factors, and special populations.

***Universal Surveillance of Severe Cases:***

Suspected case of COVID-19 requiring clinical hospitalization and/or resulting in death, or any individual with acute respiratory infection requiring hospitalization and/or resulting in death.

***COVID-19 Monitoring Units and OVR in Outpatient Settings:***

ILI (Influenza-Like Illness): Patient with acute respiratory infection, fever ≥38°C, cough, and symptom onset within 10 days. Among cases meeting the ILI case definition and tested through antigen testing, a systematic sampling is carried out for PCR testing for SARS-CoV-2, influenza, and RSV.

**During the analyzed period, the sentinel ILI strategy was integrated into the universal COVID-19 strategy in ambulatory settings. Cases were identified using the COVID-19 case definition and tested with antigen tests. For those meeting the ILI case definition, systematic sampling for molecular test was conducted.*

***Sentinel SARI Units:***

Severe Acute Respiratory Infection (SARI): Patients of any age with acute respiratory infection, fever, or a history of fever ≥38°C, cough, and symptom onset within 10 days, along with clinical requirement for hospitalization.

COVID-19 Case Definition: Available at: [https://bancos.salud.gob.ar/sites/default/files/2022-11/Actualizaci%C3%B3n%20agosto%202022%20-%20Manual%20de%20normas%20y%20procedimientos%20de%20vigilancia%20y%20control%20de%20ENO.pdf](https://nam12.safelinks.protection.outlook.com/?url=https%3A%2F%2Fbancos.salud.gob.ar%2Fsites%2Fdefault%2Ffiles%2F2022-11%2FActualizaci%25C3%25B3n%2520agosto%25202022%2520-%2520Manual%2520de%2520normas%2520y%2520procedimientos%2520de%2520vigilancia%2520y%2520control%2520de%2520ENO.pdf&data=05%7C02%7Credondolid%40paho.org%7C7300ab87b91645cd5cd308dc8c989a24%7Ce610e79c2ec04e0f8a141e4b101519f7%7C0%7C1%7C638539834279007954%7CUnknown%7CTWFpbGZsb3d8eyJWIjoiMC4wLjAwMDAiLCJQIjoiV2luMzIiLCJBTiI6Ik1haWwiLCJXVCI6Mn0%3D%7C0%7C%7C%7C&sdata=1rsdq4uWV5umLbF0llDsto%2FmxHS7d1lLXk%2Fk80wDEGE%3D&reserved=0)

ILI and SARI Case Definitions: Available at: <https://www.argentina.gob.ar/salud/coronavirus/vigilancia>, <https://bancos.salud.gob.ar/recurso/estrategia-de-vigilancia-y-control-integral-de-covid-19-y-otras-infecciones-respiratorias-0>.

1. **Chile**

***Confirmed COVID-19 Case:***

A confirmed case of COVID-19 is defined as:

(A) An individual, whether alive or deceased, with a positive PCR test for SARS-CoV-2 (SC-2).

(B) A living individual who has a positive SARS-CoV-2 (SC-2) antigen detection test conducted at a health center authorized by the Health Authority or a designated entity for administering this test.

***Influenza-like Respiratory Illness (ILI) case:*** An individual who seeks medical attention for influenza-like respiratory illness, characterized by axillary fever ≥38.5°C and cough, associated with at least one of the following symptoms: myalgias, sore throat, or headache.

***Severe Respiratory Infection (SARI) case:*** An individual who is hospitalized due to acute respiratory infection (ARI) or pneumonia without a known etiological cause and who presents with at least one of the following severity criteria:

Severity Criteria in Adults:

- Tachypnea: Respiratory rate > 26 per minute
- Hypotension: Systolic blood pressure (SBP) < 90 mmHg
- Dyspnea
- Cyanosis
- Hypoxemia: Oxygen saturation < 90% by pulse oximeter on room air
- Repeated visits due to clinical deterioration

Severity Criteria in Children:

- Hypoxemia: Oxygen saturation < 93% on room air
- Dehydration or refusal of feeds (in infants)
- Respiratory distress or increased respiratory effort
- Hemodynamic compromise
- Repeated visits due to clinical deterioration

COVID-19 Case Definition: Available at: Actualización de la definición de caso sospechoso, confirmado, probable y contacto estrecho, aislamientos y cuarentenas, medidas ante pandemia COVID-19 en Chile. ORD B51 N°269 https://www.minsal.cl/wp-content/uploads/2022/02/ORD-269-19-01-2022.pdf

ILI and SARI Case Definitions: Available at: Influenza Estacional y Pandémica. Vigilancia Epidemiológica, Investigación de Brotes y Control. Ministerio de Salud de Chile. Circular B 51/20 del 14 mayo 2010. Disponible en: http://epi.minsal.cl/wp-content/uploads/2016/04/CircularInfluenzaESTACIONALyPANDEMICA1.pdf

1. **Mexico**:

***Suspected Case of Viral Respiratory Disease:***

An individual of any age who, within the last 10 days, has presented at least one of the following signs and symptoms: cough, shortness of breath, fever, or headache*

*and*

accompanied by at least one of the following signs or symptoms: myalgia, arthralgias, sore throat, chills, chest pain, runny nose, rapid breathing, anosmia, dysgeusia, conjunctivitis

(*In children under the age of 5 years, irritability may substitute for headache.)

***Severe Acute Respiratory Infection (SARI) Case:***

Any individual who meets the criteria for a suspected case of Viral Respiratory Disease and exhibits one of the following severity indicators: shortness of breath, chest pain, or desaturation.

**Note: Suspected Case of Viral Respiratory Disease and Severe Acute Respiratory Infection were both used at sentinel and non-sentinel sites.*

Case definitions available at: Lineamiento Estandarizado para la Vigilancia Epidemiológica y por Laboratorio de la Enfermedad Respiratoria Viral: [Lineamiento Estandarizado para la Vigilancia Epidemiológica y por Laboratorio de la enfermedad respiratoria viral | Secretaría de Salud | Gobierno | gob.mx (www.gob.mx)](https://www.gob.mx/salud/documentos/lineamiento-estandarizado-para-la-vigilancia-epidemiologica-y-por-laboratorio-de-la-enfermedad-respiratoria-viral)

**Supplementary Figure 1.** Scatterplots of universal parameters vs. equivalent sentinel measures by country.

**Supplementary Figure 2.** Cross correlograms of the measures by country.
