# Supplementary figures and images for: A Comparative Analysis of Universal and Sentinel Surveillance Data for Coronavirus Disease 2019: Insights From Argentina, Chile, and Mexico (2020–2022)
Source: J Infect Dis. 2025 Mar 10;231(Suppl 2):S114–22. doi: 10.1093/infdis/jiae620 (PMC11892001; doi:10.1093/infdis/jiae620)

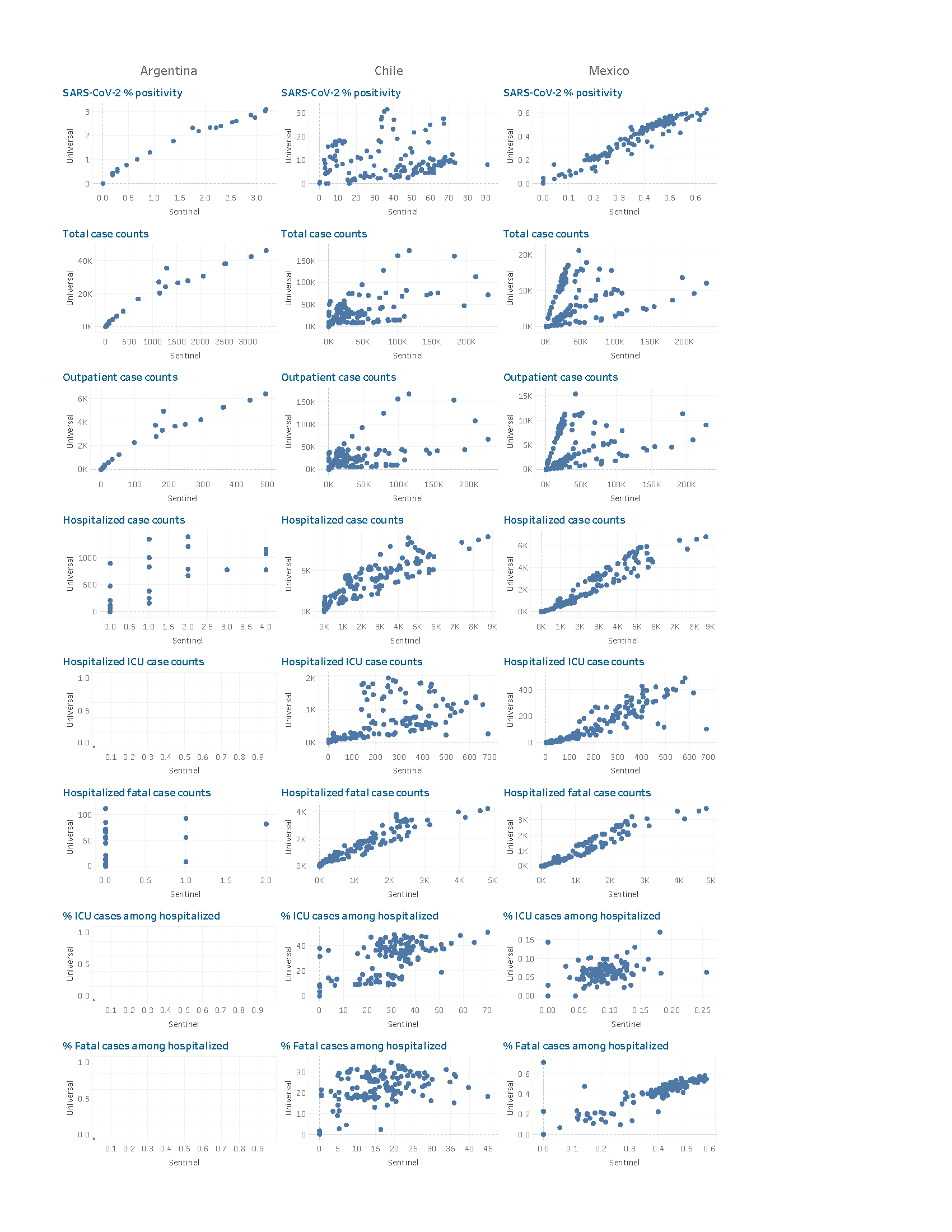

Supplement: jiae620_Supplementary_Data [file jiae620_supplementary_data.zip › SM.1 tiff.tif]

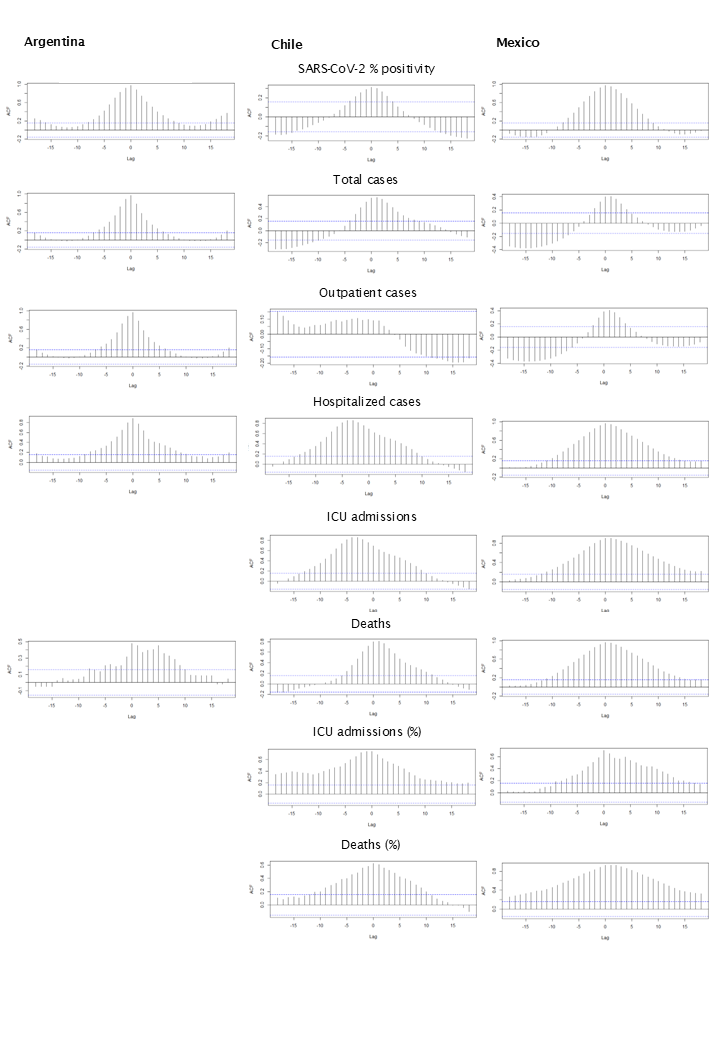

Supplement: jiae620_Supplementary_Data [file jiae620_supplementary_data.zip › SM.2 tiff.tif]
